# Supplementary material for: Unraveling the mechanisms behind the enhanced efficacy of β-lactam-based sideromycins
Source: Commun Biol. 2025 Nov 6;8:1535. doi: 10.1038/s42003-025-08898-9 (PMC12592502; doi:10.1038/s42003-025-08898-9)
Supplement: Supplementary file 2 — Supplementary Information [file 42003_2025_8898_MOESM2_ESM.pdf]

SUPPLEMENTARY INFORMATION

# Unraveling the Mechanisms Behind the Enhanced Efficacy of β-Lactam-based Sideromycins

Evelyne Lacasse[1], Renaud Binette[2], Héloïse Guibout[1], Liu Riu[3,4], Yun-Ming Lin,[3] Manuka Ghosh[3], Pierre-Luc Boudreault[5], Marvin J. Miller[3,4*], François Malouin[1**]

[1] Département de biologie, Faculté des Sciences, Université de Sherbrooke, Sherbrooke, QC, Canada.

[2] Département de chimie, Faculté des Sciences, Université de Sherbrooke, Sherbrooke, QC, Canada.

[3] Hsiri Therapeutics, Media, PA, 19063, USA.

[4] Department of Chemistry and Biochemistry, University of Notre Dame, Notre Dame, IN, USA.

[5] Département de Pharmacologie-Physiologie, Institut de Pharmacologie de Sherbrooke, Faculté de Médecine et des Sciences de la santé, Université de Sherbrooke, Sherbrooke, Québec, Canada.

## List of contents

**Table S1. Description of the strains used in this study** ........ 3

**Figure. S1. Growth of bacterial species in ID-MHBCA supplemented with iron.** ........ 4

**Table S2. Minimal inhibitory concentrations of conjugated and unconjugated β-lactams in Chelex-treated MHBCA supplemented with increasing iron concentrations.** ........ 5

**Figure. S2. Gels of purified fractions of SHV-1, AmpC, OXA-98 and PaPBP3** ........ 6

**Table S3. Kinetic parameters of purified OXA-98 from *A. baumannii* ATCC 19606.** ........ 7

**Table S4. Impact of efflux pumps inhibitors phenylalanine-arginine-β-naphthylamide (PAβN) on the activity of siderophore- β-lactams conjugates.** ........ 8

**Table S5. Impact of efflux pumps deletion mutants on the activity of conjugated and unconjugated β-lactam** ........ 9

**Table S6. Minimal inhibitory concentration (MIC) of unconjugated and conjugated β-lactams against against *E. coli* MC4100 and its hyperpermeable mutant *lptD*4213.** ........ 10

**Table S7. Percentage of each antibiotic degradation in media or bacterial supernatant at each timepoints.** ........ 11

**Table S8. Penicillin-Binding Protein assay: $IC_{50}$ values** ........ 12

**Figure S3 Uncropped and unedited gels from figures 4 and 5.** ........ 13

**Table S9. Summary of factors influencing the activity of SID-βL conjugates against different bacterial species.** ........ 14

**Table S10. List of primers used in this study** ........ 15

**Methods for the synthesis and characterization of conjugates** ........ 16

**Bis-catechol-Ampicillin (BAMP) synthesis and characterization** ........ 17
**1HNMR of BAMP** ........ 18
**13CNMR of BAMP** ........ 19
**MS of BAMP** ........ 19
**Bis-catechol-Loracarbef (BLOR) synthesis and characterization** ........ 20
**1HNMR of BLOR** ........ 21
**13CNMR of BLOR** ........ 22
**MS of BLOR** ........ 22
**Bis-catechol-mono-hydroxamate-Cefaclor (MCEF) synthesis and characterization** ........ 23
**1HNMR of MCEF** ........ 26
**MS of MCEF** ........ 26

## Table S1. Description of the strains used in this study

| Strains or plasmid | Relevant details | Source and reference |
|---|---|---|
| ***Escherichia coli*** | | |
| ATCC 25922 | Bacterial type strain | American Type Culture Collection (ATCC), Manasse, VA, USA |
| BW25113 | [F⁻ Δ(araD-araB)567 lacZ4787(Δ::*rrnB*-3) λ⁻ *rph-1* Δ (rhaD-rhaB)568 *hsdR514*] | Keio Collection[1 a] |
| BW25113Δ*tonB* | BW25113: *ΔtonB760::kan* /active transport deficient | Keio Collection [1a] |
| BW25113Δ*cir* | BW25113: *ΔcirA782::kan/* colicin and catechol | Keio Collection [1 a] |
| BW25113Δ*fepA* | BW25113: *ΔfepA721::kan*/ enterobactin transport | Keio Collection [1a] |
| BW25113Δ*fiu* | BW25113: *Δfiu777::kan/* catechol | Keio Collection [1a] |
| BW25113Δ*fhuA* | BW25113: *ΔfhuA766::kan/* hydroxamate | Keio Collection [1a] |
| BW25113Δ*fecA* | BW25113: *ΔfecA758::kan/* citric acid | Keio Collection [1a] |
| BW25113Δ*entE* | BW25113: *ΔentE732::kan/* enterobactin deficient | Keio Collection [1a] |
| MC4100 | MG1655 derivative strain [F⁻ *araD139*Δ(*argF-lac*)*U169* λ⁻ e14 *flhD5301 fruA25 relA1 rspL150 rbsR22 Δ(fimB-fimE)632(::IS1) deoC1 ptsF25*] | Laboratory collection [2] |
| MC4100 *lptD*4213 (imp4213) | MC4100 with the imp4213 allele of *lptD* lacking 23 amino acids [3]. | Laboratory collection [4] |
| MC4100 Δ*acrAB* | MC4100 with deletion of AcrAB | Laboratory collection [5] |
| BL21(DE3) | [F⁻ *ompT* $hsdS_B$ ($r_{B-}$, $m_{B-}$) *gal dcm* (DE3)] containing the pET-24b(+) plasmid [6 b] | |
| ***Klebsiella pneumoniae*** | | |
| ATCC 13883 | Also named NCTC 9633, bacterial type strain | ATCC |
| ***Pseudomonas aeruginosa*** | | |
| ATCC 27853 | Bacterial type strain | ATCC |
| PAM1020 | Wild type (PAO1 strain) | Laboratory collection [7] |
| PAM1624 | PAM1020 ΔmexCD-oprJ::Gm ΔmexEF-oprN::ΩHg | Laboratory collection [7] |
| PAM1625 | PAM1020 ΔmexAB-oprM::Cm ΔmexEF-oprN:: ΩHg | Laboratory collection [7] |
| PAM2302 | Overexpressed mexAB, mexCD and mexEF | Laboratory collection[8] |
| ***Acinetobacter baumannii*** | | |
| ATCC 19606 | Bacterial type strain | ATCC |
| **Plasmids** | | |
| pET-24b(+) | Overexpression plasmid:: $Kan^r$ | Novagen Inc. (Madison, WI, USA)[b] |

[a] Provided by Pr. Jean-Philippe Côté laboratory, Sherbrooke, Québec, Canada.
[b] Provided by Pr. Vincent Burrus Laboratory, Sherbrooke, Québec, Canada.

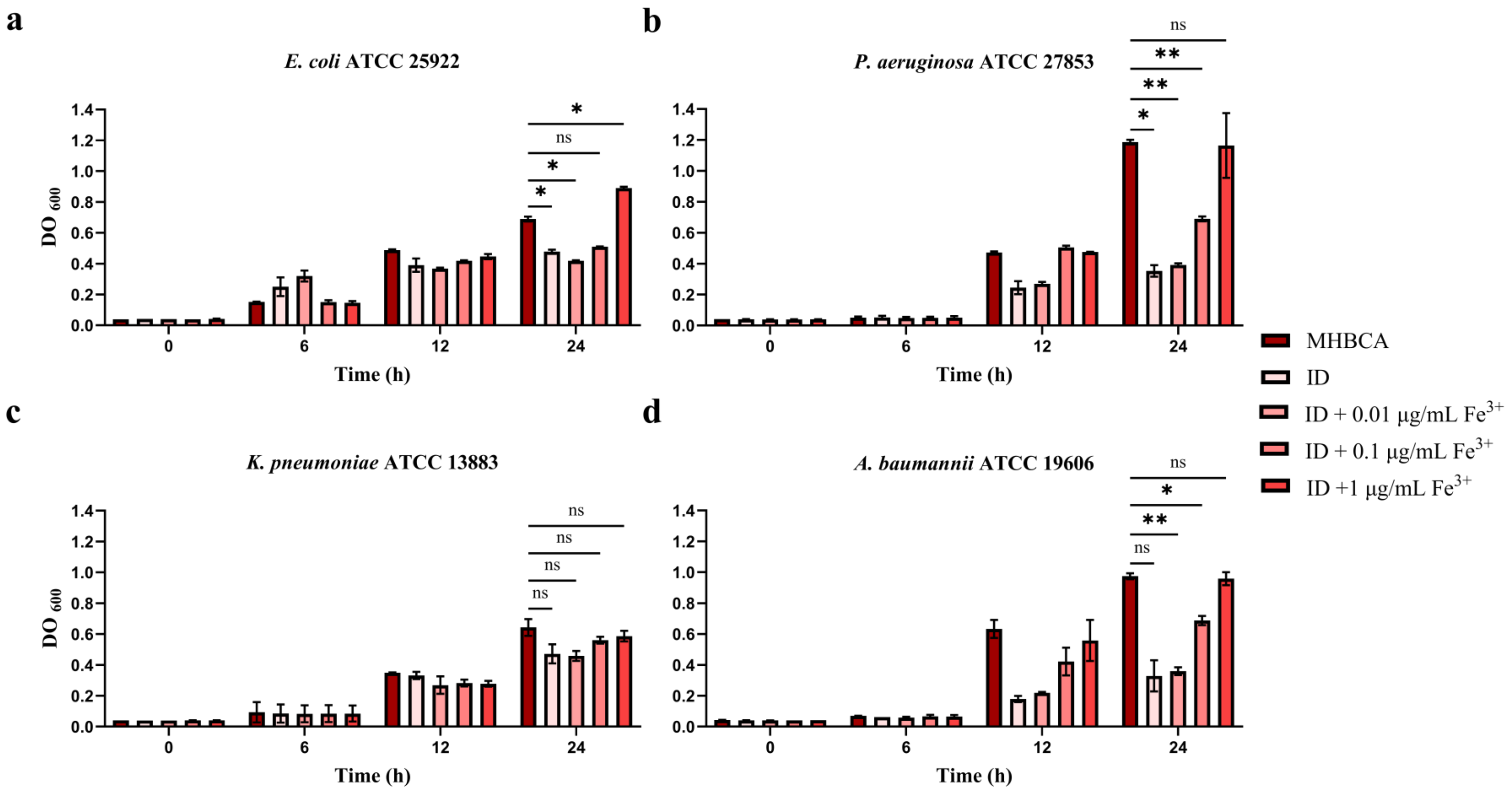


**Figure. S1. Growth of bacterial species in ID-MHBCA supplemented with iron.** Growth of all species was monitored with measurement of $OD_{600}$ at 0, 6, 12 and 24 h. All results were compiled in GraphPad Prism version 10.2.2. The growth recovery by iron complementation of iron-deprived (ID)-MHBCA was measured with the average of 4 growth curves from two biological replicates. Normality was verified with the Agostino-Pearson test for dataset at each time point. The statistical difference was measured by a two-way ANOVA and a Dunnet multiple comparison comparing the growth in MHBCA medium to ID-MHBCA with or without iron supplementation (*= p ≤0.05, **=p≤0.01) at the 24-hour point.

**Table S2. Minimal inhibitory concentrations of conjugated and unconjugated β-lactams in Chelex-treated MHBCA supplemented with increasing iron concentrations.**

| | | MICs [a] of conjugated and unconjugated β-lactam in MHBCA and in ID-MHBCA with increasing supplemented iron concentrations. | | | | | |
|---|---|---|---|---|---|---|---|
| | ID-MHBCA iron supplemented (µg/mL) and MIC ratio | AMP | BAMP | LOR | BLOR | CEF | MCEF |
| *E. coli* ATCC 25922 | 0 | 13 | 0.05 [b] | 1.6 | 0.012 | 3.1 | >200 |
| | 0.1 | ND | 0.1 | 3.1 | 0.05 | 3.1 | ND |
| | 1 | 25 | 0.2 | 3.1 | 0.1 | ND | 25 |
| | MHBCA | 13 | 0.1 | 3.1 | 0.024 | 3.1 | [6.3-25] |
| | MIC ratio 0/1 | 2 | **4** | 2 | **8** | 1 | ND |
| *K. pneumoniae* ATCC 13883 | 0 | >200 | >200 | 1.6 | 0.1 | 3.1 | >200 |
| | 0.1 | ND | ND | 1.6 | 0.4 | 3.1 | ND |
| | 1 | >200 | >200 | 3.1 | 6.3 | 6.3 | >200 |
| | MHBCA | >200 | >200 | 1.6 | 0.2 | 3.1 | >200 |
| | MIC ratio 0/1 | ND | ND | 2 | **64** | 2 | ND |
| *P. aeruginosa* ATCC 27853 | 0 | >200 | 0.1 | >200 | >200 | >200 | >200 |
| | 0.1 | ND | 0.8 | ND | >200 | ND | ND |
| | 1 | >200 | 13 | >200 | >200 | >200 | >200 |
| | MHBCA | >200 | 1.6 | >200 | 13 | >200 | >200 |
| | MIC ratio 0/1 | ND | **128** | ND | ND | ND | ND |
| *A. baumannii* ATCC 19606 | 0 | >200 | 0.2 | >200 | 0.024 | >200 | 0.2 |
| | 0.1 | ND | 0.4 | ND | 0.1 | ND | 1.6 |
| | 1 | >200 | 1.6 | >200 | 0.4 | >200 | >6.3 |
| | MHBCA | >200 | 0.4 | >200 | 0.05 | >200 | 1.6 |
| | MIC ratio 0/1 | ND | **8** | ND | **16** | ND | **>32** |

[a] MICs are given in µM, iron-deprived-MHBCA; ID-MHBCA, AMP; ampicillin, BAMP; bis-catechol-ampicillin, CEF; cefaclor, MCEF; mixed bis-catechol-mono-hydroxamate-cefaclor, LOR; loracarbef, BLOR; bis-catechol-loracarbef, ND, not determined, ID-MHBCA: iron deprived-MHBCA.

[b] In bold, are the ratio in which iron supplementation led to a dose-dependent increase in MIC leading to a ≥4-fold difference between 0 and 1 µg/mL.

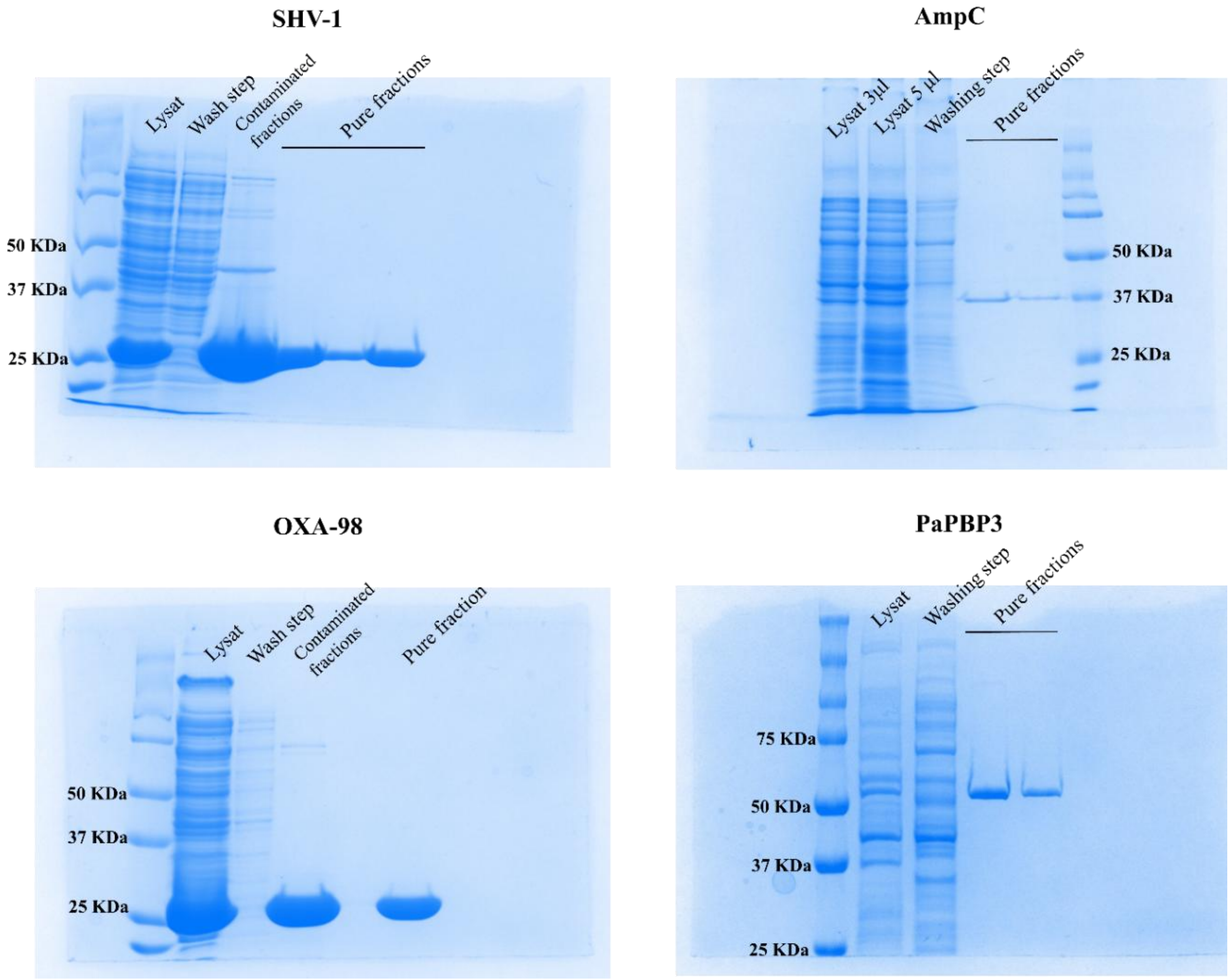


**Figure. S2. Gels of purified fractions of SHV-1, AmpC, OXA-98 and PaPBP3**. Three µl of each fraction was migrated on gel. SHV-1 appeared as a band around 25 KDa in the lysate. The band disappeared in the wash step and reappeared upon elution with imidazole firstly in contaminated fractions and secondly in pure fractions. Only fractions without any other visible band were used for further tests. The same results were obtained with OXA-98 and PaPBP3 with a band around 25 KDa and 55 KDa, respectively. The native AmpC from *P. aeruginosa* ATCC 27853 was induced with imipenem and appeared as a band around 37 KDa. The washing step with loading buffer showed a disappearance of the band and elution led to pure fractions.

**Table S3. Kinetic parameters of purified OXA-98 from *A. baumannii* ATCC 19606.**

| | OXA-98 kinetics parameters [b] | | |
|---|---|---|---|
| Antibiotic [a] | Km (µM) | Kcat ($sec^{-1}$) | Kcat/Km ($\mu M^{-1}\ sec^{-1}$) |
| LOR | NH | NH | NH |
| BLOR | NH | NH | NH |
| CEF | NH | NH | NH |
| MCEF | NH | NH | NH |
| AMP | 490 ± 12 | 22 ± 1 | 0.04 ± 0.00 |
| BAMP* | 499 ± 4 | ND | ND |
| NCF | 343 ± 10 | 3.5 ± 0.2 | 0.01 ± 0.00 |

[a] AMP; ampicillin, BAMP; bis-catechol-ampicillin, CEF; cefaclor, MCEF; mixed bis-catechol-mono-hydroxamate-cefaclor, LOR; loracarbef, BLOR; bis-catechol-loracarbef, NCF: nitrocefin. The asterisk, *, indicates that the reported parameters were measured indirectly as a Ki against nitrocefin.

[b] ND, not determined; NH, no hydrolysis detected.

**Table S4. Impact of efflux pumps inhibitors phenylalanine-arginine-β-naphthylamide (PAβN) on the activity of siderophore- β-lactams conjugates.**

| Antibiotics [b] | MICs [a] of conjugated and unconjugated β-lactam in combination with PaβN in MHBCA | | | | | |
|---|---|---|---|---|---|---|
| | *E. coli* MC4100 | | *P. aeruginosa* ATCC 27853 | | *A. baumannii* ATCC 19606 | |
| | - PaβN [c] | + PAβN | - PAβN | + PAβN | - PAβN | + PAβN |
| AMP | 13 | 13 | >200 | >200 | >200 | >200 |
| BAMP | 0.024 | 0.2 | 1.6 | 0.2 | 0.4 | >6.3 |
| | | | | | | |
| LOR | 3.1 | 6.3 | >200 | >200 | >200 | >200 |
| BLOR | 0.024 | 0.2 | 13 | >200 | 0.05 | >6.3 |
| | | | | | | |
| CEF | 6.3 | 13 | >200 | >200 | >200 | >200 |
| Mix-CEF | 6.3 | >200 | >200 | >200 | 1.6 | >6.3 |
| | | | | | | |
| ERY | 128 | 16 | >128 | 32 | ND | ND |
| RIF | ND | ND | 16 | 2 | 16 | ≤0.012 |

[a] MICs are expressed in µM except, ERY and RIF that are in µg/mL.

[b] AMP; ampicillin, BAMP; bis-catechol-ampicillin, LOR; loracarbef, BLOR; bis-catechol-loracarbef, CEF; cefaclor, MCEF; mixed bis-catechol-mono-hydroxamate-cefaclor, ERY; erythromycin, RIF; rifampicin, ND; not determined.

[c] – PaβN; without phenylalanine-arginine-β-naphthylamide, + PAβN; with 25µg/mL phenylalanine-arginine-β-naphthylamide.

**Table S5. Impact of efflux pumps deletion mutants on the activity of conjugated and unconjugated β-lactam**

| | MICs of conjugated and unconjugated β-lactam (μM) against different efflux pump mutants | | | | | |
|---|---|---|---|---|---|---|
| **Antibiotic**[a] | ***E. coli*** MC4100 | ***E. coli*** Δ*acrAB* | ***P. aeruginosa* 1020** | ***P. aeruginosa* 1624** Δ*mexCD-oprJ*, Δ*mexEF-oprN* | ***P. aeruginosa* 1625** Δ*mexAB-oprM*, Δ*mexEF-oprN* | ***P. aeruginosa* 2302** MexAB upregulated, MexCD upregulated, MexEF upregulated |
| AMP | 13 | 6.3 | >200 | >200 | >200 | >200 |
| BAMP | 0.05 | 0.05 | 0.4 | 0.4 | 0.2 | 0.4 |
| LOR | 3.1 | 3.1 | >200 | >200 | >200 | >200 |
| BLOR | 0.024 | 0.012 | 6.3 | 6.3 | 6.3 | 13 |
| CEF | 6.3 | 6.3 | >200 | >200 | >200 | >200 |
| MCEF | 6.3 | 3.1 | >100 | >100 | >100 | >100 |
| ERY | 128 | 4 | NT | NT | NT | NT |
| LVX | NT | NT | 0.5 | 0.5 | ≤ 0.06 | 16 |

[a] +; MHBCA, -; iron-deprived-MHBCA, AMP; ampicillin, BAMP; bis-catechol-ampicillin, CEF; cefaclor, MCEF; mixed bis-catechol-mono-hydroxamate-cefaclor, LOR; loracarbef, BLOR; bis-catechol-loracarbef, OXA; oxacillin, ERY; erythromycin, LVX; levofloxacin.

[b] NT: not tested.

**Table S6. Minimal inhibitory concentration (MIC) of unconjugated and conjugated β-lactams against against *E. coli* MC4100 and its hyperpermeable mutant *lptD*4213.**

| Antibiotic | MICs [a] in MHBCA (+) and ID-MHBCA (-) media | | | |
|---|---|---|---|---|
| | ***E. coli*** MC4100 | | ***E. coli*** *lptD*4213 | |
| | + | - | + | - |
| AMP | 13 | 13 | 0.4 | 0.4 |
| BAMP | 0.05 | 0.024 | 0.05 | 0.024 |
| LOR | 3.1 | 3.1 | 1.6 | 1.6 |
| BLOR | 0.012 | 0.006 | 0.006 | 0.006 |
| CEF | 6.3 | 3.1 | 3.1 | 3.1 |
| MCEF | [6.3-25] | >200 | 1.6 | 1.6 |
| VAN | >128 | NT [b] | 0.5 | NT |

[a] MICs are given in µM except VAN in µg/ml, Antibiotics: AMP; ampicillin, BAMP; bis-catechol-ampicillin, CEF; cefaclor, MCEF; mixed-cefaclor, LOR; loracarbef, BLOR; bis-catechol-loracarbef, VAN; vancomycin, +; MHBCA, -; iron-deprived (ID)-MHBCA

[b] NT: not tested.

**Table S7. Percentage of each antibiotic degradation in media or bacterial supernatant at each timepoints.**

<table>
<tr><th></th><th colspan="8">Percentage of the antibiotic [a] degradation in MHBCA (+) and ID-MHBCA (-),<br>and in [bacterial supernatant]</th></tr>
<tr><th>Time (h)</th><th>LOR</th><th>LOR</th><th>BLOR</th><th>BLOR</th><th>CEF</th><th>CEF</th><th>MCEF</th><th>MCEF</th></tr>
<tr><td></td><td>+</td><td>-</td><td>+</td><td>-</td><td>+</td><td>-</td><td>+</td><td>-</td></tr>
<tr><td>0.5</td><td rowspan="5">NDD</td><td rowspan="4">NDD</td><td>NC</td><td>NC</td><td>3</td><td>2</td><td>NC</td><td>NC</td></tr>
<tr><td>1</td><td>NC</td><td>NC</td><td>8</td><td>7</td><td>NC</td><td>NC</td></tr>
<tr><td>1.5</td><td>NC</td><td>NC</td><td>8</td><td>10</td><td>NC</td><td>NC</td></tr>
<tr><td>2</td><td>6</td><td>NDD</td><td>14</td><td>14</td><td>12</td><td>5</td></tr>
<tr><td>3</td><td>[11][b]</td><td>NC</td><td>NC</td><td>24 [36]</td><td>25 [32]</td><td>NC</td><td>NC</td></tr>
<tr><td>4</td><td>NC</td><td>NC</td><td>15</td><td>3</td><td>NC</td><td>NC</td><td>25</td><td>18</td></tr>
<tr><td>6</td><td>NC</td><td>NC</td><td>15</td><td>6</td><td>NC</td><td>NC</td><td>29</td><td>22</td></tr>
<tr><td>8</td><td>NC</td><td>NC</td><td>26</td><td>14</td><td>NC</td><td>NC</td><td>41</td><td>31</td></tr>
<tr><td>10</td><td>NC</td><td>NC</td><td>22</td><td>9</td><td>NC</td><td>NC</td><td>38</td><td>29</td></tr>
<tr><td>24</td><td>NC</td><td>NC</td><td>28</td><td>14</td><td>NC</td><td>NC</td><td>54</td><td>43</td></tr>
</table>

[a] CEF; cefaclor, MCEF; mixed bis-catechol-mono-hydroxamate-cefaclor, LOR; loracarbef, BLOR; bis-catechol-loracarbef; NC: not calculated; NDD: no degradation detected, +; MHBCA, -; iron-deprived (ID)-MHBCA

[b] In brackets are the percentage of degradation measured in the supernatant from a 4-hour bacterial culture grown in the absence of antibiotic.

**Table S8. Penicillin-Binding Protein assay: $IC_{50}$ values**

| | | $IC_{50}$ (µM) [a] | | | | | | |
|---|---|---|---|---|---|---|---|---|
| **Species** | **Antibiotic** [b] | **PBP1a** | **PBP1b** | **PBP1a/1b** | **PBP2** | **PBP3** | **PBP4** | **PBP5** |
| *E. coli* MC4100 | AMP | NA | NA | 7.22 ± 0.07 | 11.4 ± 2.4 | 1.92 ± 0.94 | 8.3 ± 0.8 | >50 |
| | BAMP* | NA | NA | 2.40 ± 0.54 | 2.26 ± 0.57 | 0.091 ± 0.007 | >50 | >50 |
| | LOR | NA | NA | 42.5 ± 11.8 | >50 | 14.9 ± 4.3 | 31.6 ± 9.0 | >50 |
| | BLOR* | NA | NA | 0.55 ± 0.02 | >50 | 0.075 ± 0.011 | 8.3 ± 0.5 | >50 |
| | CEF | NA | NA | 34.0 ± 2.2 | >50 | 15.4 ± 0.9 | 40.2 ± 8.6 | >50 |
| | MCEF* | NA | NA | 0.15 ± 0.01 | 29.3 ± 2.3 | 0.32 ± 0.01 | 8.6 ± 1.3 | >50 |
| *K. pneumoniae* ATCC 13883 | AMP | NA | NA | 8.0 ± 1.0 | 9.2 ± 1.9 | 2.6 ± 1.6 | 2.3 ± 0.31 | >50 |
| | BAMP* | NA | NA | 4.24 ± 0.07 | 5.1 ±2.1 | 0.16 ± 0.03 | 8.4 ± 2.0 | >50 |
| | LOR | NA | NA | 4.5 ± 2.1 | >50 | 4.1 ± 2.4 | 1.03± 0.72 | >50 |
| | BLOR* | NA | NA | 0.43 ± 0.17 | 33.9 ± 5.6 | 0.09 ± 0.01 | 1.7 ± 0.3 | >50 |
| | CEF | NA | NA | 7.9 ± 1.5 | >50 | 5.3 ± 2.1 | 0.74 ± 0.34 | >50 |
| | MCEF* | NA | NA | 0.35 ± 0.02 | 16.8 ± 0.3 | 0.33 ± 0.13 | 2.1 ± 0.66 | >50 |
| *P. aeruginosa* ATCC 27853 | AMP | 1.9 ± 1.5 | 2.2 ± 0.2 | NA | >50 | 0.24 ± 0.08 | ND | >50 |
| | BAMP | 1.4 ± 0.7 | 7.8 ± 1.8 | NA | >50 | 0.16 ± 0.01 | ND | >50 |
| | LOR | >50 | 1.8 ± 0.5 | NA | >50 | >50 | ND | >50 |
| | BLOR | 0.58 ± 0.04 | 1.4 ± 0.4 | NA | 4.8 ± 3.0 | 3.2 ± 0.1 | ND | >50 |
| | CEF | 33.9 ± 6.9 | 1.90 ± 0.74 | NA | >50 | 39.0 ± 1.5 | ND | >50 |
| | MCEF | 0.39 ± 0.35 | 0.83 ± 0.51 | NA | 6.74 ± 0.03 | 2.01 ± 0.48 | ND | >50 |
| *A. baumannii* ATCC 19606 | AMP | 18.0 ± 4.3 | 7.6 ± 2.0 | NA | ND | 4.5 ± 0.7 | NA | ND |
| | BAMP* | 18.5 ± 2.0 | 6.1 ± 1.7 | NA | ND | 0.42 ± 0.06 | NA | ND |
| | LOR* | >50 | >50 | NA | ND | 7.8 ± 0.8 | NA | ND |
| | BLOR* | 2.3 ± 0.7 | 5.9 ± 1.6 | NA | ND | 0.09 ± 0.01 | NA | ND |
| | CEF* | 18.0 ± 1.8 | 17.3 ± 3.5 | NA | ND | 5.4 ± 2.3 | NA | ND |
| | MCEF* | 3.1 ± 1.4 | 6.7 ± 2.9 | NA | ND | 0.27 ± 0.04 | NA | ND |

[a.] $IC_{50}$ are in µM, NA; not applicable, ND; not determined.

[b.] AMP; ampicillin, BAMP; bis-catechol-ampicillin, CEF; cefaclor, MCEF; mixed-cefaclor, LOR; loracarbef, BLOR; bis-catechol-loracarbef. Compounds with a * are a mean of a duplicate and the error is the difference between the two measurements.

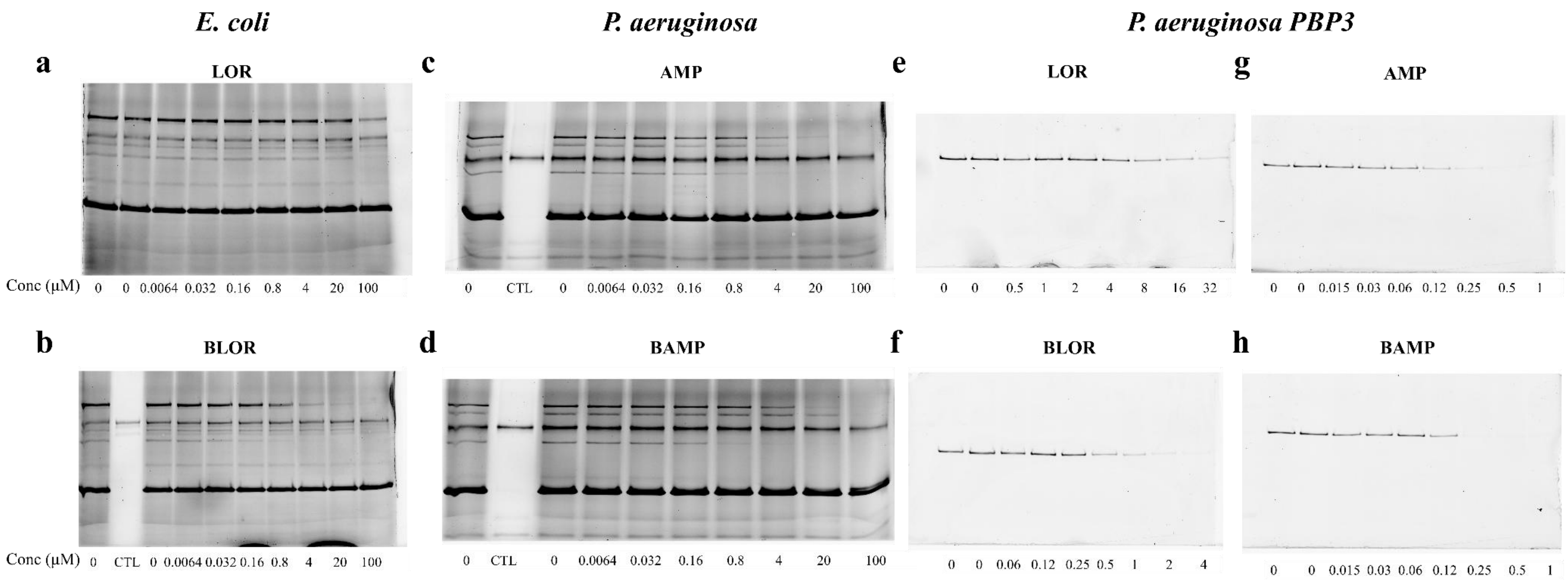


**Figure S3 Uncropped and unedited gels from figures 4 and 5**. Panel a) and b) are the gels from the membrane extract of *E. coli* MC4100. c) and d) are gels from the membrane extract of *P. aeruginosa* ATCC 27853. e), f), g) and h), are the gels from the purified *P. aeruginosa* PBP3 against loracarbef (LOR), bis-catechol-loracarbef (BLOR), ampicillin (AMP) and bis-catechol-ampicillin (BAMP) respectively.

**Table S9. Summary of factors influencing the activity of SID-βL conjugates against different bacterial species.**

| | | | | Relative impact of the different factors on observed activity of SID-βL conjugates [a] | | | |
|---|---|---|---|---|---|---|---|
| Strain | Antibiotic ratio | MIC ratio in MHBCA | MIC ratio in ID-MHBCA | Susceptibility to efflux | Resistance to β-lactamase | Outer membrane Uptake | Increased affinity to HMW PBPs |
| *E. coli* MC4100 | AMP/BAMP | 256 | 512 | O | O | ↑ | ↑↑ |
| | LOR/BLOR | 256 | 512 | O | O | ↑[b] | ↑↑↑ |
| | CEF/MCEF | ND[c] | <0.016 | O | O | ↓↓[d] | O[f] |
| *K. pneumoniae* ATCC 13883 | AMP/BAMP | No activity[e] | No activity | NT | ↓↓↓ | O | O[f] |
| | LOR/BLOR | 8 | 16 | NT | O | NT (↑) | ↑↑ |
| | CEF/MCEF | <0.016 | <0.016 | NT | ↓↓ | NT (↓)[d] | O[f] |
| *P. aeruginosa* ATCC 27853 | AMP/BAMP | >128 | >2048 | O | ↑ | NT (↑↑) | O |
| | LOR/BLOR | >16 | No activity | O | ↓↓ | NT (↑)[g] | ↑↑[g] |
| | CEF/MCEF | No activity | No activity | O | O | NT (↓↓↓) | O[f] |
| *A. baumannii* ATCC 19606 | AMP/BAMP | >512 | >1024 | NT | ↑ | NT (↑) | ↑ |
| | LOR/BLOR | >4096 | >8192 | NT | ↑ | NT (↑) | ↑↑↑ |
| | CEF/MCEF | >128 | >1024 | NT | ↑ | NT (↑) | ↑↑ |

[a] Relative impact: O, no impact; ↑, ↑↑, ↑↑↑, relative positive impact; ↓, ↓↓, ↓↓↓, relative negative impact; NT, not tested; (↑or↓), hypothetical impact.

[b] Slow and steady uptake of BLOR correlating with a sustained bactericidal effect.

[c] ND, not determined due to the Eagle effect. Growth was inhibited in the concentration range of 6.3-25 µM.

[d] MCEF uptake is limited and endogenous siderophore(s) like enterobactin seems to prevent its activity in ID-MHBCA.

[e] MICs were >200 µM for both unconjugated and conjugated β-lactams in the indicated medium.

[f] An increased affinity for HMW PBPs was measured, but had no impact on the conjugate activity due to other factors (e.g., β-lactamase susceptibility or uptake).

[g] β-lactamases reduced BLOR activity in MHBCA and ID-MHBCA, however its MIC in MHBCA is due to an increased affinity for HMW PBPs and maybe to an efficient uptake.

**Table S10. List of primers used in this study**

| Primers | Sequence (5' – 3') | Function |
|---|---|---|
| Gibs_pET_N1 | CATATGTATATCTCCTTCTTAAAGTTAAAC | Linearized pET-24b(+) without the T7 tag. |
| Gibs_pET_X1 | GCACTCGAGCACCAC | Linearized pET24b(+) with 6xHis-tag |
| Gibs_SHV_N2 | TAAGAAGGAGATATACATATGCCGCAGCCGCTTGAGCAA | Amplified SHV-1 from *Klebsiella pneumoniae* without signal peptide for Gibson assembly |
| Gibs_SHV_X1 | GTGGTGGTGCTCGAGTGCGCGTTGCCAGTGCTCG | Amplified SHV-1 from *Klebsiella pneumoniae* without stop codon for Gibson assembly |
| Gibs_OXA_N2 | TAAGAAGGAGATATACATATGAAATCTGATGAAAAAGCAGAG | Amplified OXA-98 from *Acinetobacter baumannii* without signal peptide for Gibson assembly |
| Gibs_OXA_X1 | GTGGTGGTGCTCGAGTGCTAAAATACCTAATTGTTCTAAGCT | Amplified OXA-98 from *Acinetobacter baumannii* without stop codon for Gibson assembly |
| Gibs_PBP3_N2 | TAAGAAGGAGATATACATATGGTGCGGCATATAGCCATCCC | Amplified a soluble from *Pseudomonas aeruginosa* PaPBP3 for Gibson assembly |
| Gibs_PBP3_X1 | GTGGTGGTGGTGCTCGAGGCCACGCCCTCCTTTTGC | Amplified PaPBP3 from *Pseudomonas aeruginosa* without stop codon for Gibson assembly |

**Methods for the synthesis and characterization of conjugates**

All solvents and reagents were obtained from commercial sources and used without further purification unless otherwise stated. Isobutyl chloroformate ($ClCO_2iBu$) was used from Acros Seal anhydrous bottles. Technical grade tetrahydrofuran (THF) was freshly distilled over sodium before use. OmniSolv® LCMS water from EMD Millipore was used for all reactions, purifications, and work-up purposes. Reactions were conducted under an atmosphere of dry argon unless otherwise stated. Commercially available cefaclor was purchased from Sigma Aldrich.  Lorabid was a gift from Eli Lilly and Co.

Sorbent Technologies silica gel 60 (32−63 μm) was used for column chromatography purifications. Reverse-phase chromatographic purifications were performed on Teledyne Instruments' 30 g RediSep Rf Gold® C18Aq reversed-phase columns (column volume: 26.4 ml, average particle size: 20–40 µm, average pore size: 100 Å) at a flow rate of 35 ml $min^{-1}$. Thin layer chromatography (TLC) was performed with Al-backed Merck 60-F254 or Al-backed Merck RP-C18 F256 silica gel plates using a 254 nm lamp and aqueous $FeCl_3$ for visualization. HPLC–MS mass measurements were used to determine purity and structural integrity. All characterized compounds were determined to be ≥95% pure. The HPLC–MS studies were performed with a Bruker MicrOTOF-Q II Quadrupole Time-of-Flight mass spectrometer operating in positive ion mode with an acquisition mass range of 50–3000 u. Electrospray ionization source parameters were capillary voltage = 2200 v, end slate offset = −500 v, nebulizer gas pressure = 5 bar, dry gas flow rate = 10 l $min^{-1}$, and dry gas temperature = 220 °C. Liquid separation was performed on a Dionex UltiMate 3000 RSLC with mobile phases consisting of water with 0.1% formic acid (A) and acetonitrile with 0.1% formic acid (B). The mobile phase gradient at 0.4 ml $min^{-1}$ was 10% of B for 2 min followed by a linear ramp of 100% of B at 18 min and a return to initial conditions from 18.1 to 20 min. UV–VIS spectra were recorded on a Dionex UltiMate 3000 RS Diode Array Detector over the 190–400 nm wavelength range. The LC column was a Thermo Scientific Acclaim RSLC 120 C18 with 2.2 µm particle size, 120 Å pore size, and 2.1 × 100 mm dimensions heated at 40 °C. $^{1}H$ spectra of the compounds were recorded on a Varian DirectDrive 600 spectrometer operating at a proton resonance frequency of 599.98 MHz.

**Bis-catechol-Ampicillin (BAMP) synthesis and characterization**

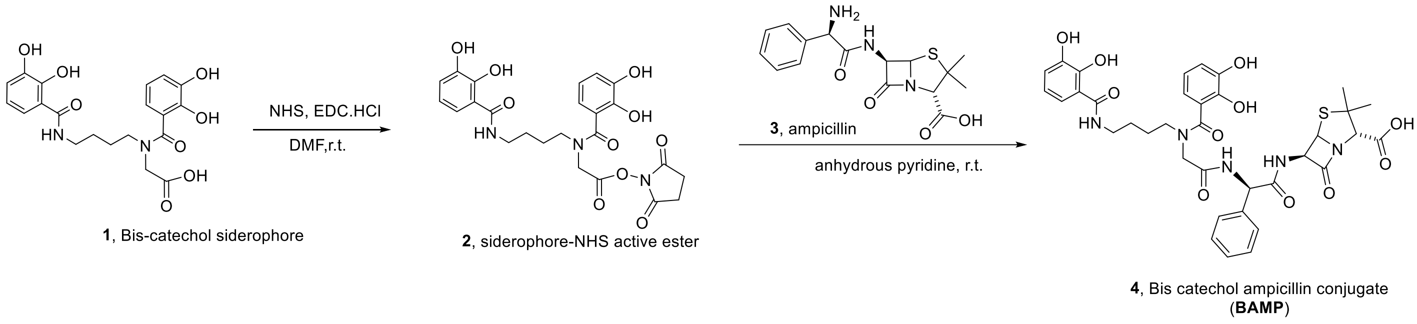


**(4) (2*S*,6*R*)-6-((*R*)-2-(2-(*N*-(4-(2,3-dihydroxybenzamido)butyl)-2,3-dihydroxybenzamido)acetamido)-2-phenylacetamido)-3,3-dimethyl-7-oxo-4-thia-1-azabicyclo[3.2.0]heptane-2-carboxylic acid)**. To a solution of the bis-catechol siderophore free acid (**1**, 0.5 mmol, 209 mg) in 5 mL of anhydrous DMF was added EDC·HCl (0.6 mmol, 115 mg) and NHS (0.55 mmol, 63 mg), the mixture was stirred at room temperature overnight. When the starting siderophore was completely converted to the NHS active ester (**2**), ampicillin (**3**, 0.5 mmol, 174 mg) was added to the above mixture followed by anhydrous pyridine (1 mmol, 79 μL). The reaction was stirred overnight and monitored by LC/MS. After the reaction was finished, the reaction mixture was directly loaded onto a C18 chromatography column and eluted with 30% acetonitrile in water to give siderophore-ampicillin (20 mg, 0.027 mmol) conjugate (**4**, **BAMP**) as a white solid in 54% yield after lyophilization. $^{1}$H-NMR (400 MHz, DMSO-$d_6$) δ 0.97 (t, J = 8.0 Hz, 1H), 1.11-1.20 (m, 1H), 1.26-1.31 (m, 2H), 1.4 (s, 3H), 1.44-1.45 (m, 1H), 1.53 (s, 3H), 1.60-1.63 (m, 2H), 1.91 (s, 1H), 2.47 (s, 1H), 2.65-2.66 (s, 1H), 2.95-3.0 (m, 1H), 3.09-3.16 (m, 2H), 3.30-3.37 (m, 2H), 3.88-3.98 (m, 1H), 4.12-4.24 (m, 1H), 5.31 (d, J = 4.0 Hz, 1H), 5.43 (brs, 1H), 5.65-6.03 (m, 1H), 6.44-6.56 (m, 1H), 6.65-6.66 (m, 1H), 6.77 (t, J = 8.0 Hz, 1H), 6.88 (d, J = 8.0 Hz, 1H), 7.27-7.38 (m, 5H), 7.44 (d, J = 8.0 Hz, 1H), 8.58-8.65 (m, 1H), 8.70-8.82 (m, 1H), 9.08-9.17 (m, 1H). $^{13}$C-NMR (400 MHz, DMSO-$d_6$) δ 16.14, 21.60, 25.68, 26.71, 27.49, 31.25, 34.55, 37.24, 43.52, 55.64, 55.91, 58.09, 64.51, 67.38, 72.68, 115.45, 117.57, 118.25, 119.17, 127.35, 127.45, 128.02, 128.66, 138.42, 138.60, 141.75, 145.88, 146.73, 150.22, 158.68, 168.65, 169.78, 170.10, 172.54, 173.53. HRMS calculated for C36H40N5O11S (M+H$^+$) 750.24395; found 750.24481.

1HNMR of BAMP

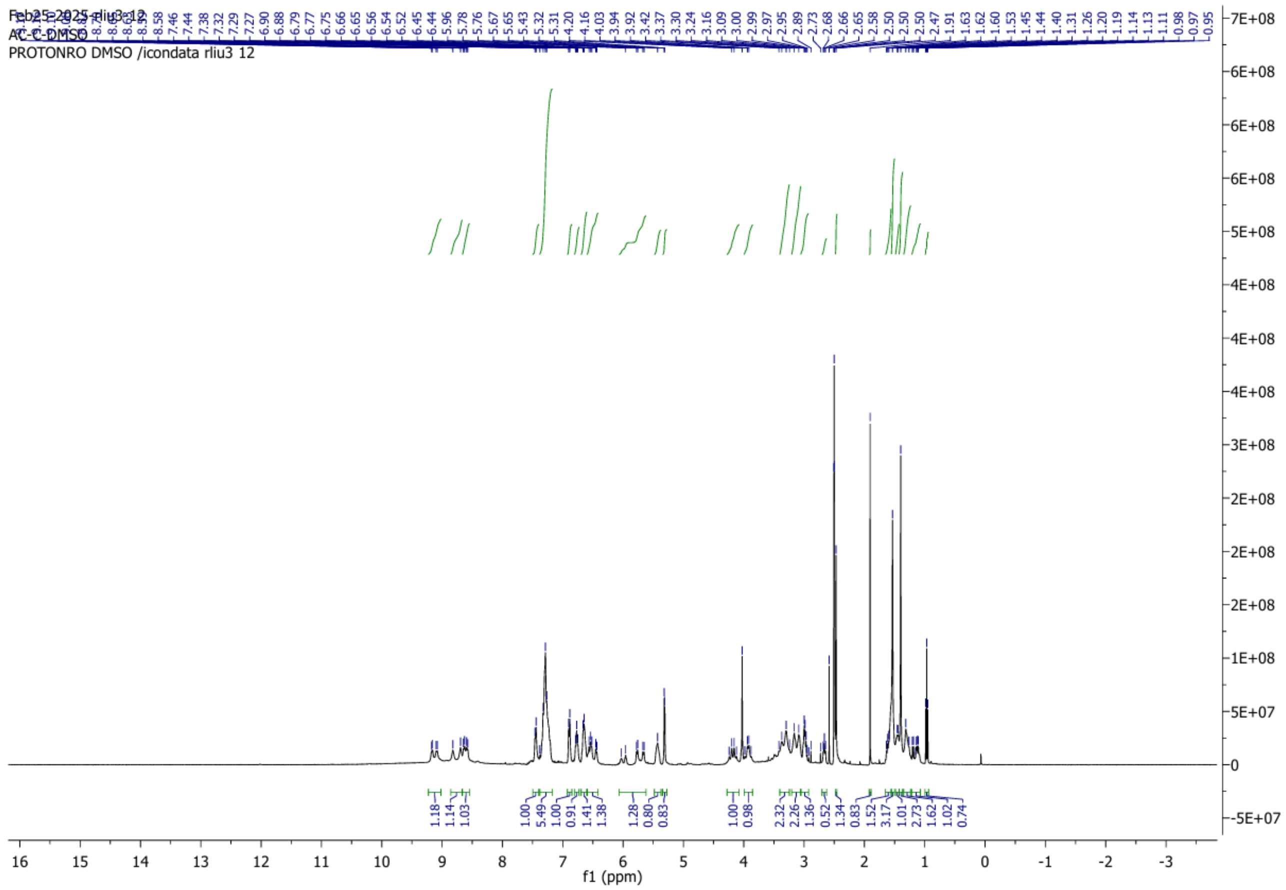

## 13CNMR of BAMP

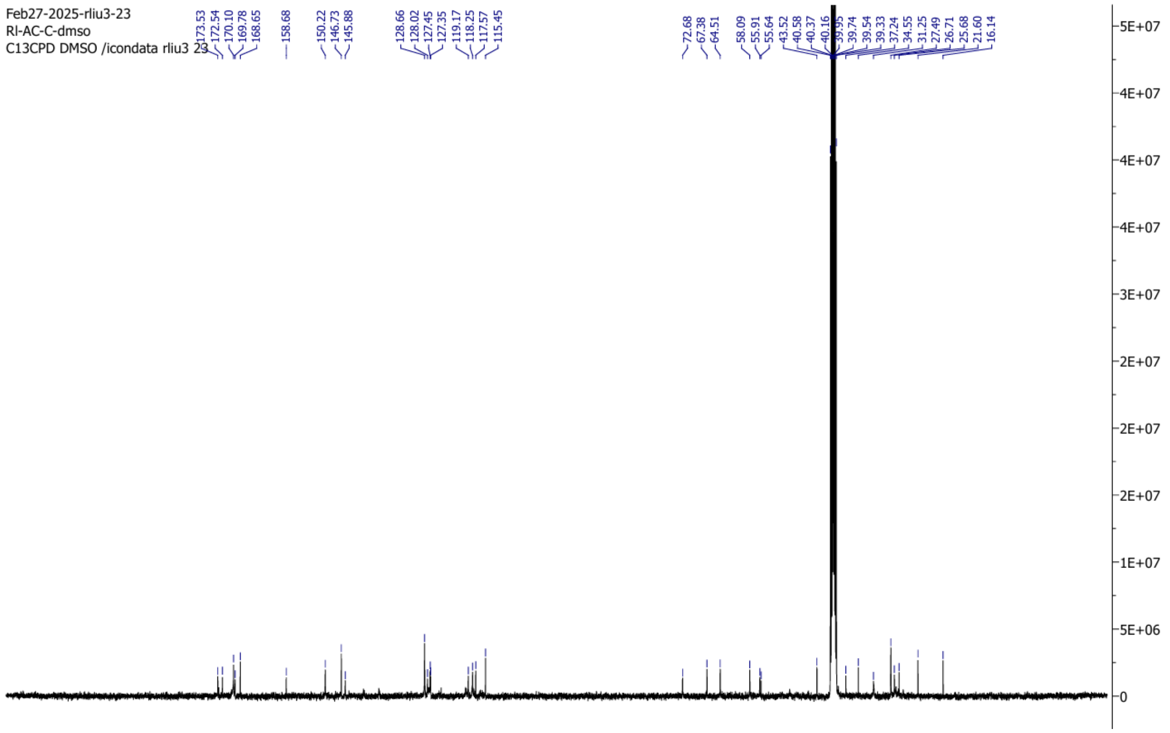


## MS of BAMP

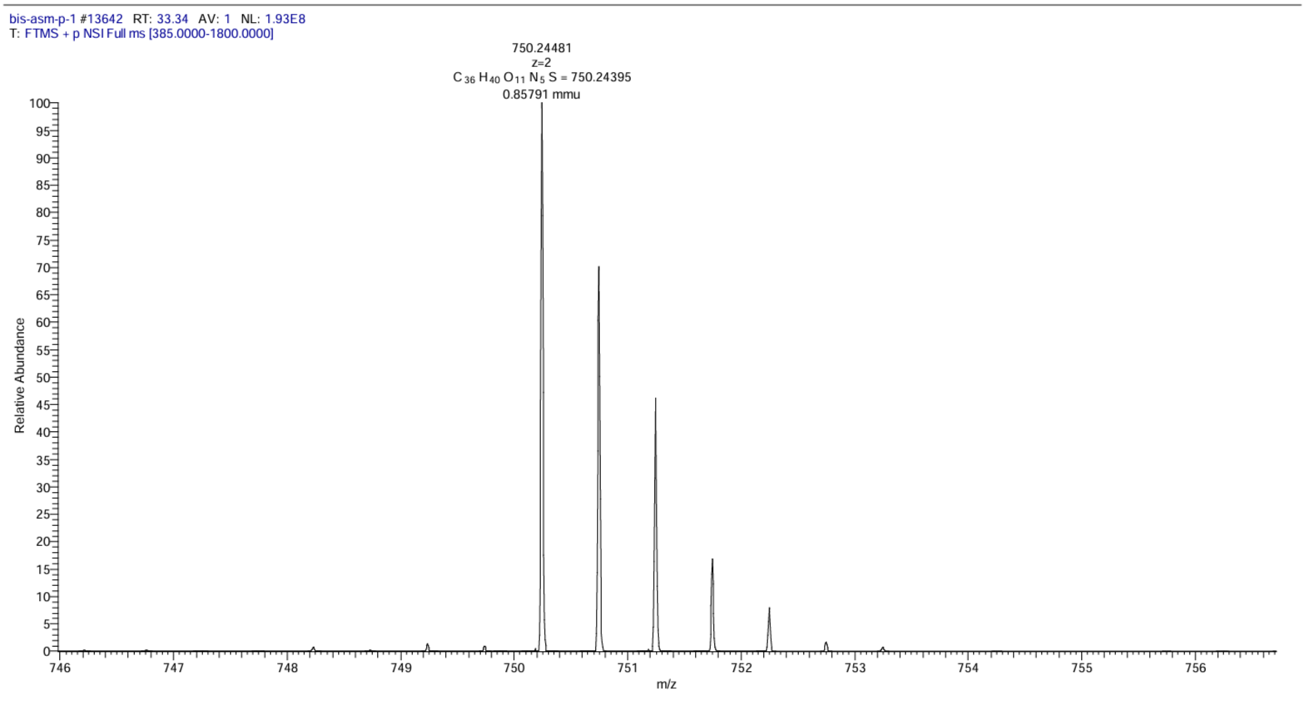

**Bis-catechol-Loracarbef (BLOR) synthesis and characterization**

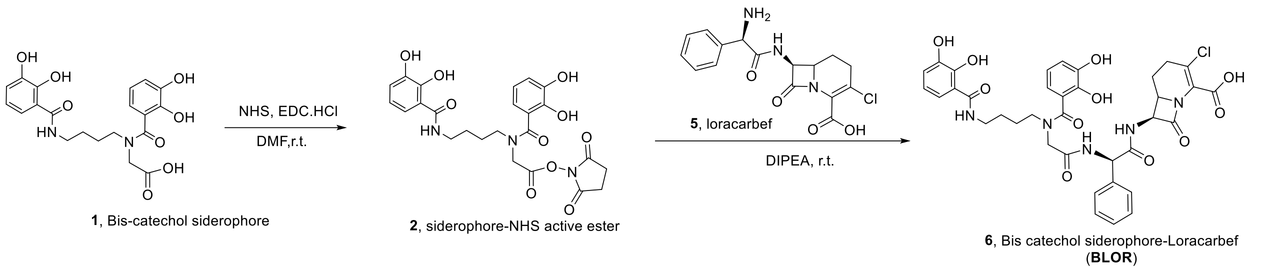


**(6)**. **(7*S*)-3-chloro-7-((*R*)-2-(2-(*N*-(4-(2,3-dihydroxybenzamido)butyl)-2,3-dihydroxybenzamido)acetamido)-2-phenylacetamido)-8-oxo-1-azabicyclo[4.2.0]oct-2-ene-2-carboxylic acid**. To a solution of the bis catechol siderophore free acid (**1**, 0.5 mmol, 209 mg) in 5 mL of anhydrous DMF was added EDC·HCl (0.6 mmol, 115 mg) and NHS (0.55 mmol, 63 mg), the mixture was stirred at room temperature overnight. When the starting siderophore was completely converted to NHS active ester (**2**), Loracarbef (**5**, 0.5 mmol, 174 mg) was added to the above mixture followed by anhydrous DIPEA (1 mmol, 184 µL). The reaction was stirred overnight and monitored by LC/MS. After the reaction was finished, the reaction mixture was directly loaded onto a C18 chromatography column and eluted with 30% acetonitrile in water to give bis catechol - Loracarbef conjugate (**BLOR**, **6**, 15 mg, 0.02 mmol) as a white solid in 40% yield after lyophilization. $^1$H-NMR (400 MHz, DMSO-$d_6$) δ 0.97 (t, J = 8.0 Hz, 1H), 1.31-1.39 (m, 2H), 1.46-1.56 (m, 4H), 1.91 (s, 1H), 2.21-2.25 (m, 1H), 2.41 (s, 2H), 2.57 (s, 1H), 2.97-3.00 (m, 1H), 3.09-3.16 (m, 2H), 3.24-3.31 (m, 2H), 3.59-3.65 (m, 1H), 3.94 (s, 1H), 4.12-4.27 (m, 1H), 5.19 (s, 1H), 5.43-5.56 (m, 1H), 5.94-6.03 (s, 1H), 6.44-6.53 (m, 1H), 6.57-6.68 (m, 1H), 6.74-6.79 (m, 1H), 6.88 (d, J = 8.0 Hz, 1H), 7.22-7.35 (m, 5H), 7.43 (s, 1H), 8.61-8.88 (m, 2H), 9.16 (s, 1H). $^{13}$C-NMR (400 MHz, DMSO-$d_6$) δ 16.15, 21.58, 22.14, 26.99, 28.58, 30.17, 34.54, 37.39, 43.95, 51.05, 52.10, 55.98, 56.87, 57.61, 115.62, 117.73, 118.22, 119.13, 125.55, 127.50, 128.21, 128.82, 145.83, 146.72, 150.15, 158.66, 163.21, 164.04, 168.87, 169.76, 170.03, 172.53. HRMS calculated for C36H37ClN5O11 (M+H$^+$) 750.21726; found 750.21729.

# 1HNMR of BLOR

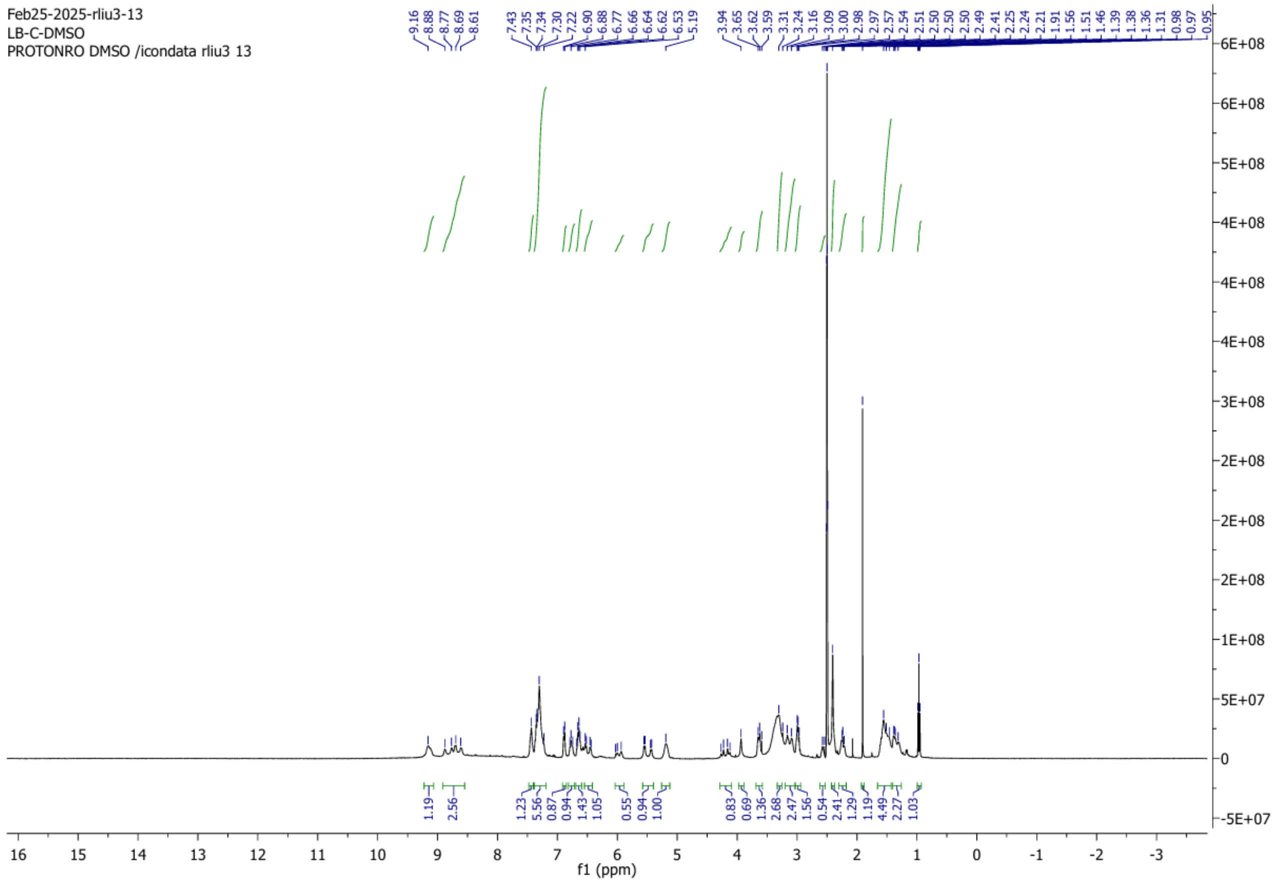

# 13CNMR of BLOR

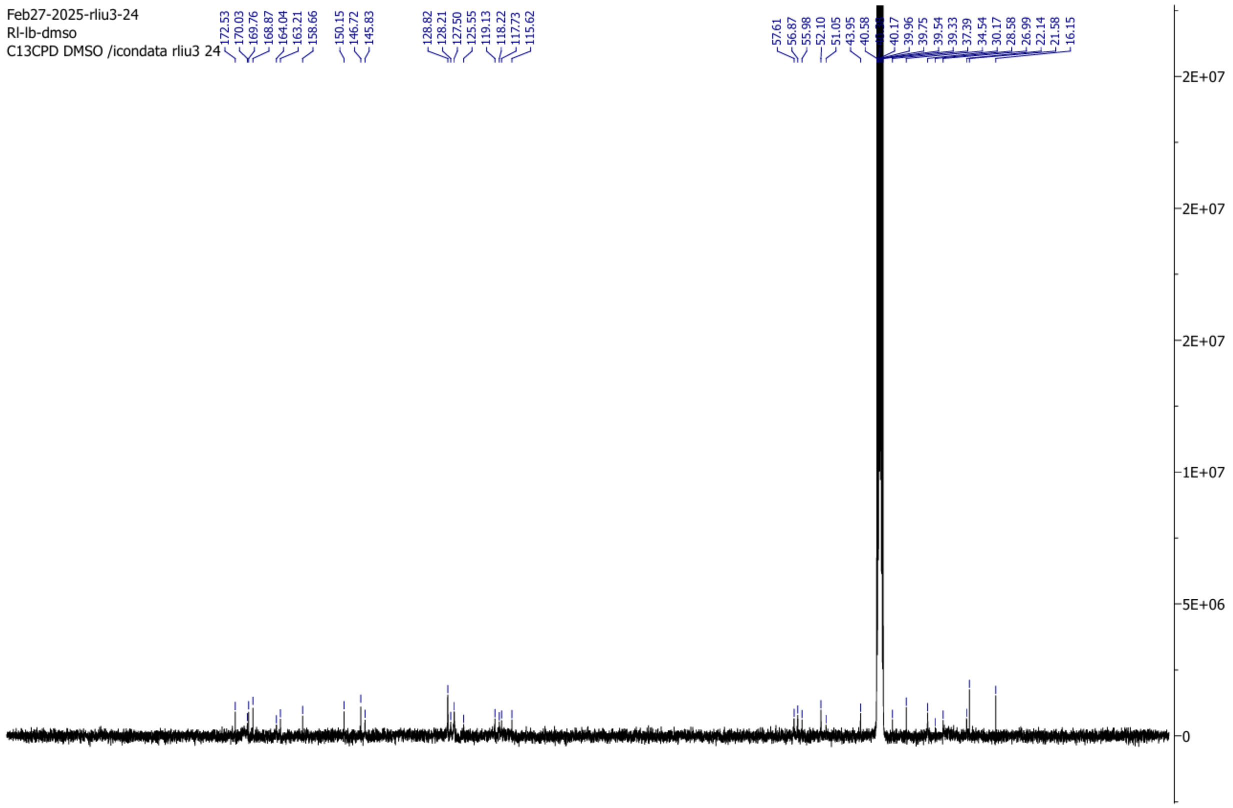


# MS of BLOR

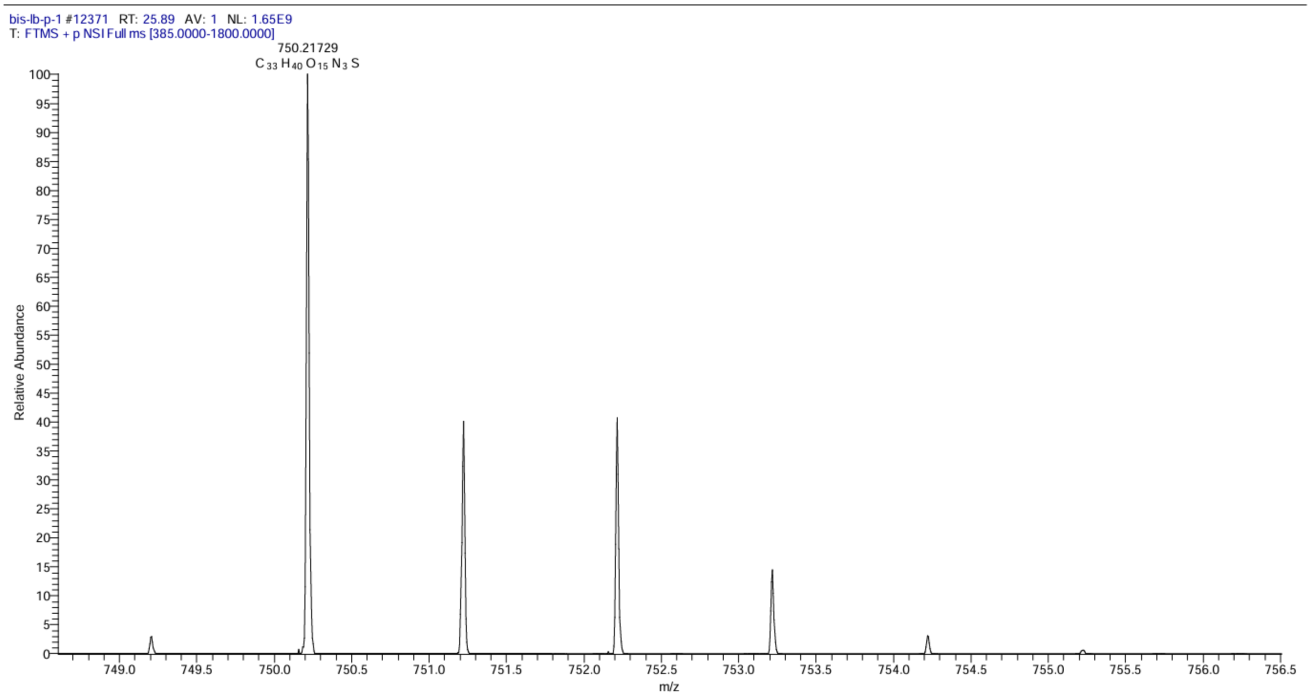

## Bis-catechol-mono-hydroxamate-Cefaclor (MCEF) synthesis and characterization

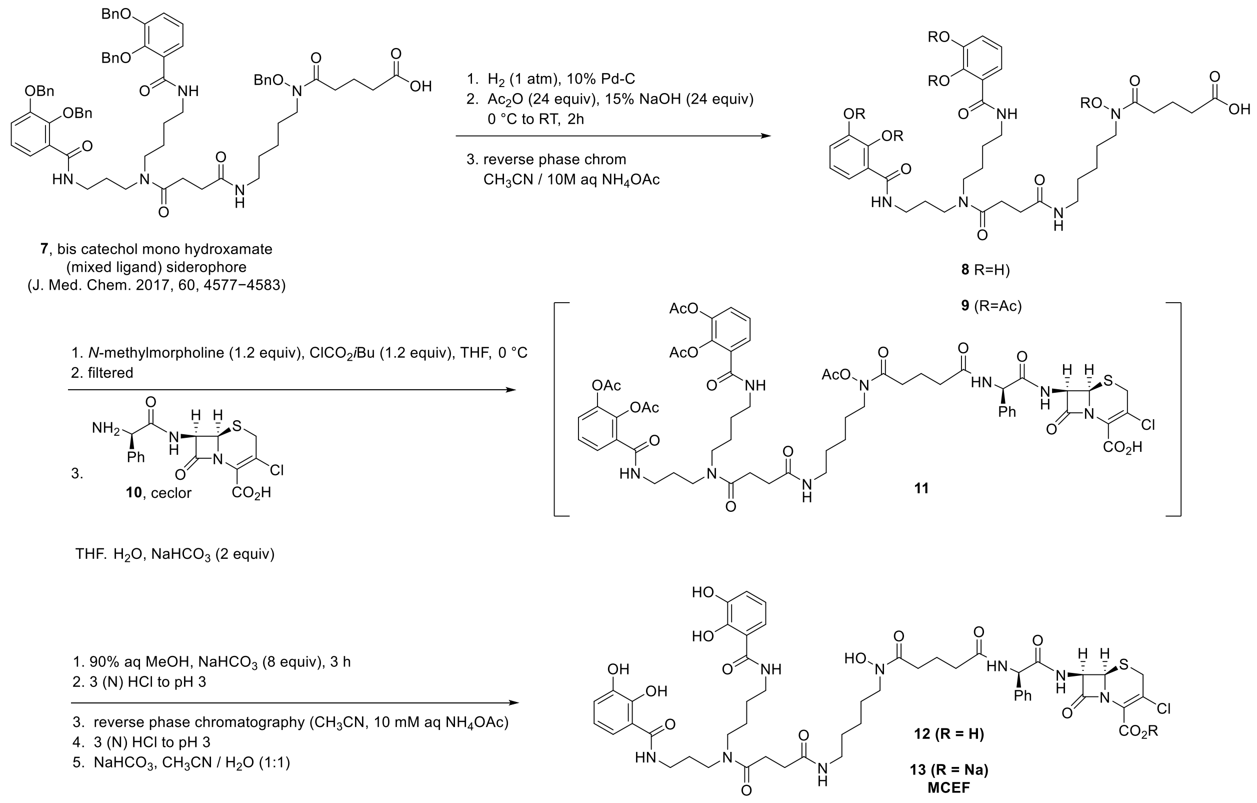


**(7) 7-(3-(2,3-dihydroxybenzamido)propyl)-1-(2,3-dihydroxyphenyl)-18-hydroxy-1,8,11,19-tetraoxo-2,7,12,18-tetraazatricosan-23-oic acid.** The pentabenzyl-protected mixed ligand siderophore analog **7** (1.50 g, 1.23 mmol) of fimsbactin was dissolved in 50 mL of MeOH. The solution was purged with argon, charged with 10% Pd-C (225 mg) and then subjected to catalytic hydrogenolysis under a hydrogen atmosphere (1 atm) for 10 h. The reaction mixture was again purged with argon, filtered, and washed with MeOH, and the solvent was evaporated at room temperature to give the deprotected mixed ligand **8** (0.9g, 100 % yield) that was used directly for the next reaction. $^1$H NMR (DMSO-d6, 600 MHz) δ 1.16–1.18 (m, 2H), 1.32–1.37 (m, 2H), 1.44–1.59 (m, 5H), 1.64–1.69 (m, 2H), 1.76–1.82 (m, 1H), 2.21 (t, J =7.2Hz, 2H), 2.26–2.31 (m, 2H), 2.35 (t, J= 6Hz, 2H), 2.47–2.51 (m, 2H), 2.94–2.97 (m, 2H), 3.15–3.32 (m, 10H), 3.41–3.45 (m, 2H), 6.62–6.66 (m, 2H), 6.86–6.88 (m, 2H, ArH), 7.20–7.26 (m, 2H, ArH), 7.74–7.76 (m, 1H, NH), 8.73–8.82 (m, 2H, NH). LCMS (m/z): $[M+H]^+$ calcd for $C_{35}H_{49}N_5O_{12}$, 732.3404; found, 732.3450.

**(9) 18-acetoxy-7-(3-(2,3-diacetoxybenzamido)propyl)-1-(2,3-diacetoxyphenyl)-1,8,11,19-tetraoxo-2,7,12,18-tetraazatricosan-23-oic acid**. A solution of **8** (0.9 g, 1.23 mmol), NaOH (1.18 g, 29.5 mmol,

24 equiv) in $H_2O$ (8 mL) containing a few ice chips was added $Ac_2O$ (2.8 mL, 29.5 mmol, 24 equiv). The reaction mixture was allowed to warm to room temperature while stirring over 2 h, then cooled to 0 °C (ice bath), acidified with 3N HCl to pH 3, and extracted with ethyl acetate (3 x 10 mL). The combined extracts were washed with water (5 mL), brine (5 mL), dried ($MgSO_4$), filtered, and concentrated under reduced pressure. The residue was purified using reverse-phase column chromatography eluting with a gradient (10-50%) of $CH_3CN/NH_4OAc$ (10 mM) to provide the pentaacetyl siderophore **9** as a fluffy white solid (927 mg, 80%) after lyophilization. $^1H$ NMR ($CD_3OD$ in $CDCl_3$, 600 MHz) δ 1.13–1.83 (m, 12H), 2.18–2.28 (m, 19H), 2.30–2.36 (m, 2H), 2.51–2.54 (m, 2H), 2.94–3.66 (m, 14H), 7.15–7.21 (m, 2H), 7.34–7.36 (m, 2H), 7.41–7.46 (m, 2H). LCMS (m/z): $[M+H]^+$calcd for $C_{45}H_{60}N_5O_{17}$, 942.3979; found, 942.3960.

**(12) (6R,7R)-3-chloro-7-((R)-7-(3-(2,3-dihydroxybenzamido)propyl)-1-(2,3-dihydroxyphenyl)-18-hydroxy-1,8,11,19,23-pentaoxo-25-phenyl-2,7,12,18,24-pentaazahexacosan-26-amido)-8-oxo-5-thia-1-azabicyclo[4.2.0]oct-2-ene-2-carboxylic acid**. To a cooled (0 °C, ice-bath) solution of pentaacetyl-protected bis-catechol-N-hydroxamate siderophore **9** (2.5 g, 2.68 mmol, obtained from repetitions of the synthesis of **9**) and *N*-methyl morpholine (NMM, 354 µL, 3.22 mmol, 1.2 equiv) in anhydrous THF (30.0 mL) was added and $ClCO_2$-*i*Bu (417 µL, 3.22 mmol, 1.2 equiv). A white precipitate formed gradually after adding the chloroformate (*N*-methylmorpholine hydrochloride salt). The reaction mixture was stirred under an atmosphere of argon for 1 h at 0 °C and the progress of the isobutyl formate ester formation was monitored by TLC analysis for complete consumption of starting siderophore **9.**

To the above reaction mixture was added a solution of Cefaclor (**10**, 1 g, 2.94 mmol, 1.1 equiv) and $NaHCO_3$ (0.54 g, 6.46 mmol, 2.2 equiv) and the reaction was stirred at 0 °C for 1 h, then at room temperature for 2 h. THF was evaporated from the reaction mixture, and the residue was dissolved in ETOAc (100 mL). The combined organic layer was washed with water and brine, then dried over $Na_2SO_4$, filtered and concentrated under reduced pressure to afford crude **11** as a white solid (3.5 g) in near quantitative yield.

To a solution of crude compound **11** (3.5g) in 40mL of 90% aq. MeOH was added, solid $NaHCO_3$ (1.78 g, 0.021 mmol, 8 equiv) and the reaction was stirred for 3 h monitoring by LCMS analysis until complete deacetylation. The reaction mixture was filtered to remove excess $NaHCO_3$. After evaporation of MeOH, the residue was dissolved in 25mL of $CH_3CN$: $H_2O$ (1:1) and maintained at pH 3 with 3N HCl to

precipitate the crude conjugate **12**. Conjugate **12** was further purified in batches (4 x 500 mg) by four consecutive reverse phase chromatographies (RediSep Rf Gold reversed-phase C18 high-performance columns, 30 g) using a gradient of 10−50% of $CH_3CN/NH_4OAc$ (10 mM) as the eluent. The desired fractions collected at 20−30% of $CH_3CN/NH_4OAc$ (10 mM) from four batches were combined and lyophilized to give the poly ammonium salt of **12**. The isolated salt form of conjugate **12** was dissolved in $CH_3CN/H_2O$ (1:1, 10 mL), acidified with 3 N HCl to pH 3, concentrated to remove $CH_3CN$, and lyophilized to provide the neutral conjugate **12** (1 g, 35%); $^1$H NMR (600 MHz, DMSO-d6) δ 1.16-1.18 (m, 2H), 1.31-1.81 (m, 6H), 2.19-2.33 (m, 3H), 2.47-2.48 (m, 10H), 2.95-2.96 (m, 2H), 3.20-3.43 (m, 9H), 3.65 (d, *J* = 18 Hz, 1H), 4.90 (d, *J* = 4.8 Hz, 1H), 5.47-5.49 (m, 1H), 5.65 (d, *J* = 8.4 Hz, 1H), 6.61-6.68 (m, 2H), 6.87-6.88 (m, 2H), 7.22-7.40 (m, 5H), 7.75-7.76 (m, 1H), 8.49 (d, *J* = 8.4 Hz, 1H), 8.80-8.92 (m, 2H), 9.25 (d, *J* = 8.4 Hz, 1H). LCMS for $C_{50}H_{61}ClN_8O_{15}S$: calcd 1080.37, found 1081.3843($MH^+$).

**(13, MCEF ) Sodium (6R,7R)-3-chloro-7-((R)-7-(3-(2,3-dihydroxybenzamido)propyl)-1-(2,3-dihydroxyphenyl)-18-hydroxy-1,8,11,19,23-pentaoxo-25-phenyl-2,7,12,18,24-pentaazahexacosan-26-amido)-8-oxo-5-thia-1-azabicyclo[4.2.0]oct-2-ene-2-carboxylate**. Conjugate **12** (1 g, 0.92 mmol) was dissolved in $CH_3CN/H_2O$ (1:1, 25 mL) and the solution was treated with $NaHCO_3$ (85 mg, 1.01 mmol) in water (3 mL, LCMS grade). The resulting solution was lyophilized after evaporation of $CH_3CN$ to provide the desired Na salt **13**, in quantitative yield; $^1$H NMR (600 MHz, DMSO-d6) δ 1.16-1.18 (m, 2H), 1.31-1.81 (m, 6H), 2.19-2.33 (m, 3H), 2.48-2.51 (m, 10H), 2.94-2.96 (m, 2H), 3.20-3.43 (m, 9H), 3.65 (d, *J* = 18 Hz, 1H), 4.91 (d, *J* = 6 Hz, 1H), 5.47-5.49 (m, 1H), 5.65 (d, *J* = 8.4 Hz, 1H), 6.64-6.71 (m, 2H), 6.87-6.90 (m, 2H), 7.27-7.42 (m, 5H), 7.76-7.77 (m, 1H), 8.48-8.49 (d, *J* = 8.4 Hz, 1H), 8.80-8.91 (m, 2H), 9.24 (d, *J* = 8.4 Hz, 1H). LCMS for $C_{50}H_{61}ClN_8O_{15}SNa$: calcd 1102.35, found 1081.3554 ($MH^+$-Na), 1103.3355 ($MH^+$+Na).

## 1HNMR of MCEF

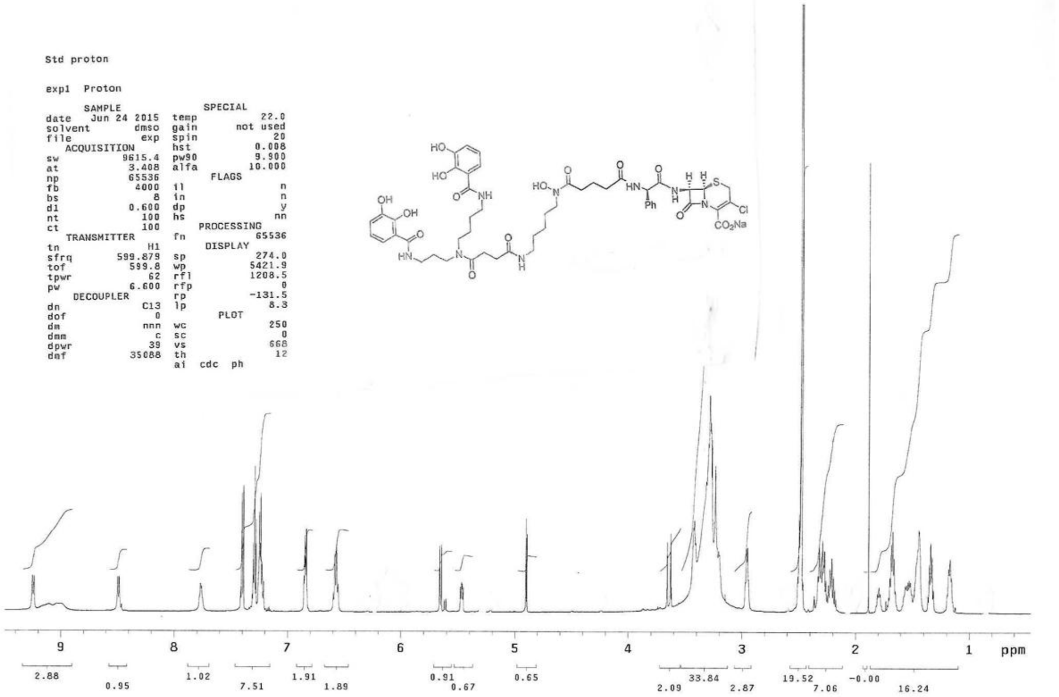


## MS of MCEF

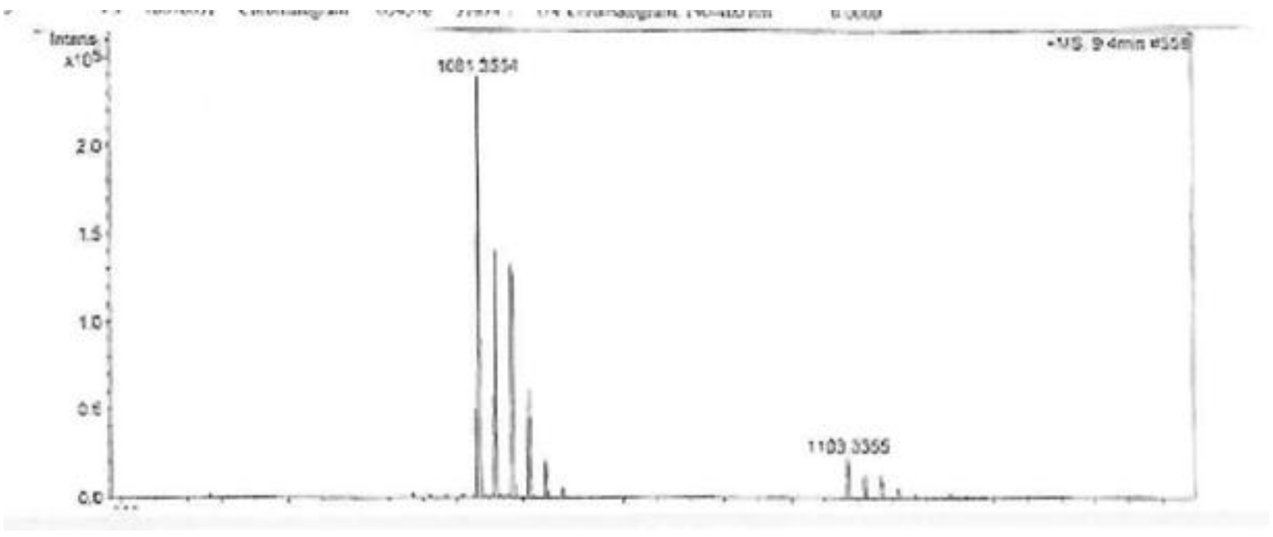

## References


1. Baba, T. *et al.* Construction of *Escherichia coli* K-12 in-frame, single-gene knockout mutants: the Keio collection. *Mol Syst Biol* (2006) doi:10.1038/msb4100050.

2. Casadaban, M. J. Transposition and Fusion of the *lac* Genes to Selected Promoters in *Escherichia coli* using Bacteriophage Lambda and Mu. *J Mol Biol* **104**, 541–555 (1976).

3. Braun, M. & Silhavy, T. J. Imp/OstA is required for cell envelope biogenesis in *Escherichia coli*. *Molecular Microbiology* **45**, 1289–1302 (2002).

4. Sampson, B. A., Misra, R. & Benson, S. A. Identification and Characterization of a New Gene of *Escherichia coli* K-12 Involved in Outer Membrane Permeability. *Genetic* **122**, 491–501 (1989).

5. Gerken, H. & Misra, R. Genetic evidence for functional interactions between TolC and AcrA proteins of a major antibiotic efflux pump of *Escherichia coli*. *Mol Microbiol* **54**, 620–631 (2004).

6. Studier, F. W. & Moffatt, B. A. Use of bacteriophage T7 RNA polymerase to direct selective high-level expression of cloned genes. *J Mol Biol* **189**, 113–130 (1986).

7. Lomovskaya, O. *et al.* Use of a Genetic Approach To Evaluate the Consequences of Inhibition of Efflux Pumps in *Pseudomonas aeruginosa*. *Antimicrob Agents Chemother* **43**, 1340–1346 (1999).

8. Bostion, K., Glinka Tomasz, Lomovskaya, O. & Surber, M. *Use and administration of Bacterial efflux pump inhibitors*. **WO Patent 2005/089738** (2005).
