# Supplementary material for: Unraveling the mechanisms behind the enhanced efficacy of β-lactam-based sideromycins
Source: Commun Biol. 2025 Nov 6;8:1535. doi: 10.1038/s42003-025-08898-9 (PMC12592502; doi:10.1038/s42003-025-08898-9)
Supplement: Supplementary file 3 — Description of Additional Supplementary Files [file 42003_2025_8898_MOESM3_ESM.pdf]

Description of Additional Supplementary Files

File name- Supplementary Data 1

File description – Data for figure 3.
